# A Cell-Based Small Molecule Screening Method for Identifying Inhibitors of Epithelial-Mesenchymal Transition in Carcinoma

**DOI:** 10.1371/journal.pone.0033183

**Published:** 2012-03-14

**Authors:** Kian-Ngiap Chua, Wen-Jing Sim, Victor Racine, Shi-Yun Lee, Boon Cher Goh, Jean Paul Thiery

**Affiliations:** 1 Cancer Science Institute of Singapore, National University of Singapore, Singapore, Singapore; 2 Institute of Molecular and Cell Biology, A*STAR (Agency for Science, Technology and Research), Singapore, Singapore; 3 Experimental Therapeutics Centre, A*STAR (Agency for Science, Technology and Research), Singapore, Singapore; 4 Department of Haematology-Oncology, National University Hospital, Singapore, Singapore; The University of Kansas Medical Center, United States of America

## Abstract

Epithelial Mesenchymal Transition (EMT) is a crucial mechanism for carcinoma progression, as it provides routes for *in situ* carcinoma cells to dissociate and become motile, leading to localized invasion and metastatic spread. Targeting EMT therefore represents an important therapeutic strategy for cancer treatment. The discovery of oncogene addiction in sustaining tumor growth has led to the rapid development of targeted therapeutics. Whilst initially optimized as anti-proliferative agents, it is likely that some of these compounds may inhibit EMT initiation or sustenance, since EMT is also modulated by similar signaling pathways that these compounds were designed to target. We have developed a novel screening assay that can lead to the identification of compounds that can inhibit EMT initiated by growth factor signaling. This assay is designed as a high-content screening assay where both cell growth and cell migration can be analyzed simultaneously via time-course imaging in multi-well plates. Using this assay, we have validated several compounds as viable EMT inhibitors. In particular, we have identified compounds targeting ALK5, MEK, and SRC as potent inhibitors that can interfere with EGF, HGF, and IGF-1 induced EMT signaling. Overall, this EMT screening method provides a foundation for improving the therapeutic value of recently developed compounds in advanced stage carcinoma.

## Introduction

Epithelial Mesenchymal Transition (EMT) is a fundamental process driving embryonic development, particularly during gastrulation and in morphogenesis of the heart primordium, neural crest and somites [1]-[3]. Cells engaged in the EMT program undergo complex changes in cell architecture and behavior. In a typical epithelial layer, epithelial cells develop adhesive structures between adjacent cells, such as adherens junctions, desmosomes and tight junctions, to establish robust intercellular adhesions. Epithelial cells are apico-basal polarized, with the apical and basal surfaces serving different functions. Mesenchymal cells, on the other hand, do not have stable intercellular junctions and possess front-to-back leading edge polarity. These characteristics also increase the migratory capacity in mesenchymal cells, owing to the shift of weaker cell-cell adhesion and stronger cell-matrix adhesion. Thus, the EMT program describes a series of events during which epithelial cells lose many of their epithelial characteristics and take on properties that are typical of mesenchymal cells.

For more than a decade, EMT has been recognized as a potential mechanism for the progression of carcinoma [4]-[5]. At the onset of tumor progression, dysregulation of the cell cycle machinery can result in proliferation of the normal epithelia to give rise to an adenoma. The adenoma, with additional genetic and epigenetic alterations, can later progress to a carcinoma *in situ*. The carcinoma *in situ* is believed to engage the EMT program at the micro-invasive stage [5]-[6], allowing individual carcinoma cells to migrate and intravasate into lymph and blood vessels and eventually disseminate and metastasize to distant organs.

Metastasis of the primary tumor is assisted by the release of cytokines and growth factors that are secreted by the surrounding stroma [7]-[9]. Cancer patients are reported to have elevated serum levels of growth factors, namely hepatocyte growth factor (HGF), epidermal growth factor (EGF), transforming growth factor-beta (TGF-β) and insulin-like growth factor-1 (IGF-1), among others. In addition, numerous carcinoma are found to have over-expression of either wild-type or mutated kinases [10]-[11]. These kinase oncogenes play important roles in growth factor signal transduction regulation, and their dysregulation can lead to survival and excessive proliferation of cancer cells as well as the initiation and sustenance of the EMT program and tumor metastasis [12]-[14]. These findings have generated great interest in understanding the role of oncogenes and their signaling cascades in tumor growth and the EMT program.

The discovery of oncogene addiction in sustaining tumor growth has led to the development of modern molecular targeted therapeutics [15]. These small molecule inhibitors function by binding to the ATP-binding site of the dysregulated kinase oncogene, thereby inhibiting the phosphorylation and activation of its signal transduction cascade responsible for sustaining tumor growth. Many preclinical studies have showed the effectiveness of targeted small molecule inhibitors in killing cancer cells or preventing tumor growth. Examples include Imatinib Mesylate for the treatment of chronic myeloid leukemia [16], and Gefitinib for the treatment of non-small-cell lung cancer [17]. Whilst originally identified and optimized for their anti-proliferative effects, evidence suggests that some of these targeted small molecule inhibitors may also inhibit EMT initiation or sustenance, since the EMT program is modulated by similar signaling pathways for which these molecules have been generated [9], [18]. For example, Ki26894, an ALK5 inhibitor, has recently been shown to decrease the invasiveness and EMT of scirrhous gastric cancer cells [19]. However, an extensive screening effort to identify and quantify the relative effectiveness of existing targeted small molecule inhibitors in EMT modulation has not been methodically attempted.

In this paper, we present the design and development of a novel EMT inhibition drug screening assay (spot migration assay) using a model carcinoma reporter cell line (NBT-II), which can be induced to undergo EMT initiated by different growth factors: EGF, HGF, or IGF-1. This assay allows us to identify the EMT modulating properties of targeted small molecule compounds by inhibiting EMT signaling in response to growth factor treatment.

## Materials and Methods

### Preparation of compound stock plates

Test compounds were purchased from various vendors (Selleck Chemicals, Sigma Aldrich, SYN|thesis MedChem, and Tocris Bioscience). Compound stocks were assembled in 96-well V-bottom plates (Greiner). For screening studies, test compounds at both 0.25 mM and 1.0 mM concentrations in DMSO were prepared, with each occupying a single well in columns 2–11 of the stock plates. For dose response studies, test compounds were prepared in duplicate wells and serially diluted in DMSO, starting with a 1.0 mM concentration. Stock plates were stored at −20^o^C and thawed to room temperature before use.

### Spot migration assay

Nara Bladder Tumor No. 2 (NBT-II) cells were purchased from American Type Culture Collection (ATCC) and were stably transfected with mcherry-fluorescent H2B. Cells were maintained in DMEM supplemented with 10% fetal bovine serum (FBS, HyClone Thermo Scientific), 1 µg/ml puromycin (Sigma) and 100 units/ml penicillin-streptomycin (1× pen-strep, Invitrogen). Cells were grown to 80% confluency in tissue culture flasks prior to plating. Cells were trypsinized and concentrated to a density of 5×10^6^ cell/ml in CO_2_-independent medium (Invitrogen) supplemented with 10% FBS. The cell suspension was then evenly dispensed into the wells of 2 columns of a 96-well V-bottom plate. Using a robotic liquid-handling station (Bravo, Agilent Technologies), 0.5 µl of cell suspension was transferred from the 2 columns of the cell suspension-loaded plate and deposited into the center of the wells of 2 columns of a blank 96-well polystyrene tissue-culture treated clear bottom, black assay plate (Corning #3904). This process was repeated six times so that all 96 wells of the assay plate were deposited with a cell suspension spot. The plate was then sealed to minimize evaporation of the cell suspension spots and transferred to a 37^o^C, 5% CO_2_ incubator to allow for cells to attach to the culture surface. After 1 h, the plate was gently washed with medium once to remove unattached cells, refreshed with 100 µl of assay medium (DMEM supplemented with 10% FBS and 1× pen-strep), and then further incubated to allow for cell-cell contacts to establish in the cell colonies.

After 4 h of incubation, the cell colonies for each well were imaged using a confocal microplate imager (MetaXpress Ultra, Molecular Devices) with 10×Plan Fluor objective, 561 nm laser excitation and 593/40 nm emission filter configuration. Four tiled, non-overlapping images were acquired around the center of each well, and were then stitched together during image analysis to generate a montage covering an area of 3.2 mm×3.2 mm. These images (T1) represent the initial state of the cell colonies before EMT induction.

After the T1 images were acquired, 1 µl of test compounds were transferred from compound stock plates and added to the assay plates. Appropriate negative controls (1 µl DMSO) and positive controls (1 µl 1.0 mM compound in DMSO) were also added into columns 1 and 12 of each assay plate, respectively. The assay was optimized to use AG1478, JNJ-38877605 and BMS-536924 as reference positive control compounds for EGF-, HGF- and IGF-1-induced EMT, respectively. The cultures were then further incubated overnight.

The next day, 50 µl of growth factor-containing medium was added to each well of the assay plates. For each of the growth factor-induced EMT spot migration assays, we optimized the final growth factor concentrations in each well to be 20 ng/ml EGF (Sigma), 4 ng/ml HGF (Calbiochem) or 150 ng/ml IGF-1 (R&D Systems), respectively. The cultures were then incubated for another 24 h, to allow for EMT and sufficient cell motility/dispersion to occur in the cell colonies.

Finally, the cell colonies were imaged again using the microplate imager, as described above. These images (T2) represent the final state of the cell colonies after compound treatment and EMT induction. The acquired T1 and T2 image sets for each assay plate were then sent for image analysis.

### Image analysis routine

The acquisition of each well was obtained by four adjacent field images with no interstice in between. Each field image was loaded and nuclei were initially segmented independently of other fields to prevent artifact at the field border. Nuclei segmentation was achieved by combining a wavelet transform which is robust to noise and inhomogeneous background, and a watershed algorithm based on intensities to split nuclei clusters. Mask of nuclei segmentation of the different fields were then stitched together in order to obtain a large segmentation of the whole well. Cell bodies were estimated by applying a morphological dilation on the nucleus segmentation using a disk of 30 pixels (48 µm) in diameter. In cells that were contiguous, such as in a cell colony, nuclei segmentation dilation will result in the formation of continuous region areas with surrounding cells which can then be identified as colonies. In general, a well will contain one big colony (corresponding to the initial cell spot) and several much smaller colonies (corresponding to single cells, small cluster of cells, dust and contamination). Only nuclei contained in the biggest colony were kept and subsequently analyzed. A spreading coefficient was derived from nuclei positions in order to measure how much the colony had dispersed. The spreading coefficient is defined as the standard deviation of the cell positions in the cell colony relative to the center of the colony:
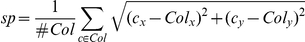
where *Col* indicates all cells of a colony, *#Col* is the total cell number, [*Col_x_, Col_y_*] is the average position of all nuclei in the colony, [*c_x_, c_y_*] is the position of the cell c. The coefficient *sp* is homogenous with a distance and indicates the relative cell dispersion from the colony center.

Total cell number and spreading coefficient values for each well were then exported into an Excel sheet. The Cell Count Ratio (CCR) and Cell Dispersion Ratio (CDR) values were calculated by combining data calculated from T1 and T2 images for each well, while the normalized CDR or CDR% of each well calculated by taking the CDR values of negative and positive controls as the boundary limits:
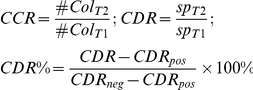
where *CDR_pos_* and *CDR_neg_* are the average CDR values of the negative and positive control wells respectively in each test plate [20].

### EMT time-lapse video

EMT inhibitory effects of selected compounds were validated by NBT-II epithelial colony time-lapse videoscopy. NBT-II cells were plated onto a 12-well plate (BD) at a low density of 2,000 cells per well in 1 ml of assay medium. Cells were allowed to grow and form epithelial colonies for a period of 72 h. The cultures were then refreshed with assay medium containing test compounds and further incubated overnight. The next day, growth factor (EGF, HGF or IGF-1) was added prior to video imaging. Video imaging of individual cell colonies was performed using a video microscope incubator system (Axiovert-200M, Carl Zeiss). Time-lapse images were taken at 5 min intervals for 19 h.

### Western blots

NBT-II cells were treated with compounds at 0.5, 2 and 8 µM overnight and incubated with a growth factor for 24 h. Cells were lysed with protease/phosphatase inhibitor-containing RIPA buffer. Proteins were separated in 8% polyacrylamide gels and transferred to PVDF membranes. Membranes were blocked in 5% BSA and incubated at 4^o^C overnight with MMP-13 (Millipore), E-cadherin (BD) and α-tubulin (Sigma) primary antibodies. Membranes were then developed with HRP-conjugated secondary antibody (Amersham) and ECL substrate (Millipore).

### Statistical analysis

Error bars in CDR dose response plots represent standard deviation of replicate samples. CDR IC_50_ values were calculated through sigmoidal curve fitting of CDR dose response plots using GraphPad Prism software.

## Results

### EMT spot migration assay design and assembly

**Figure 1:**
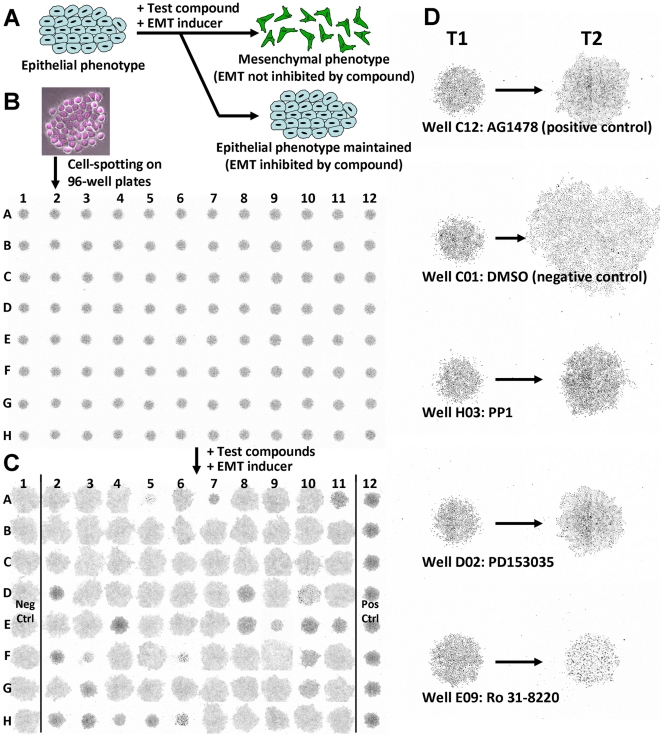
EMT spot migration screening assay overview. (A) Schematic of the spot migration screening assay to identify EMT inhibitory compounds. EMT can be initiated and maintained in epithelial cells via growth factor signaling. This assay measures the dispersion of cells in the presence of a test compound and an EMT inducer (EGF, HGF or IGF-1). The prevention of cell dispersion directly correlates to the propensity of a test compound to block an induced EMT signaling pathway. (B) Screening assay image acquisition workflow. Robot-assisted plating of H2B-mcherry transfected NBT-II cells into the well centers of 96-well plates. The initial plate image acquired at T1 served as the baseline reference for calculating the CCR and CDR values for each well. The cells were treated with test compounds overnight and further incubated for 24 h with a growth factor to induce EMT. (C) Final plate image acquired at T2 depicted the dispersion response of cells 24 h after addition of the compounds and growth factor treatment. In the example shown, columns 2–11 were treated with 80 different test compounds at 6.67 µM and EGF. Column-1 served as negative controls treated with 0.67% DMSO and EGF, while column-12 served as positive controls treated with 6.67 µM AG1478 and EGF. (D) Magnified images of selected wells acquired at T1 and T2. Wells C12, H03 and D02 are examples of cell colonies treated by compounds that inhibited EGF-initiated cell dispersion and did not inhibit cell growth. Well C01 is a cell colony undergoing EGF-induced EMT without any dispersion inhibition. Well E09 is a cell colony treated by a growth inhibitory or toxic compound.

An overview of the EMT screening assay is illustrated in Figure 1. Our primary motivation for this assay was to screen for compounds that have the propensity to inhibit EMT in cells induced by exogenous growth factor signaling (Figure 1A). To achieve this, we designed a high-throughput method of forming compact, consistent epithelial cell colonies in 96 wells. Using a multi-channel liquid-handling machine, a 0.5 µl high density suspension of cells was directly deposited into the center of wells of 96-well plate, in a highly consistent manner (Figure 1B). As the cell suspension drop was initially deposited onto a dry surface, the cells were confined within the drop, and will be kept confined until they firmly attach. Thus, the drop area essentially determined the boundary of the initial epithelial colony that will be formed in the well. As the environmental conditions essential for effective cell attachment were 37^o^C and 5% CO_2_, the plate was sealed to prevent the 0.5 µl drop from evaporating in the incubator. In addition, CO_2_-independent medium was used instead of normal culture media to compensate for the lack of circulating CO_2_ in the sealed plate. This sealed environment can be maintained for more than an hour for cell attachment to be completed, after which the wells can be gently washed and refreshed with normal culture media. Due to the initial compactness, the attached cells can quickly establish cell-cell contacts and consistent epithelial colonies will form hours after cell plating.

In this study, we used the NBT-II reporter cell line. The cells were stably transfected with H2B-mcherry to label the nuclei, so that the migration of these cells could be tracked through live-cell fluorescent imaging. NBT-II is an ideal cell model for the study of EMT because of its fast EMT phenotypic response to several known EMT stimuli, such as EGF, HGF and IGF-1 [21]-[22]. As evident in Figure 1C, a complete EMT cell motility response was achieved within 24 h after the addition of an EMT stimulus. This short response time is critical for our screening assay design, as the EMT quantification may be masked by cell proliferation response if the motility response is too slow.

For compound screening, we first optimized the spot migration assay to identify compounds that could inhibit EMT induced by EGF, HGF, or IGF-1 signaling. Appropriate compounds (AG1478, JNJ-38877605 and BMS-536924) were selected as reference positive controls for each EGF, HGF and IGF-1 EMT assay, respectively. The screen was conceptualized as a high-content imaging assay, whereby colony nuclei in each well were imaged and analyzed prior to compound treatment (T1 images), and 24 h after EMT induction (T2 images). The effect of the screening compounds in this assay were grouped into three categories: (1) compounds that are cytotoxic or growth inhibitory to cells; (2) compounds that can inhibit EMT, and are not growth inhibitory to cells (our desired compound property); and (3) compounds that are not EMT or growth inhibitory. The cell colony examples shown in Figure 1D highlighted these three different categories. The grouping of compounds into these three categories was determined through image analysis of the plate images.

### Image analysis and assay robustness

**Figure 2:**
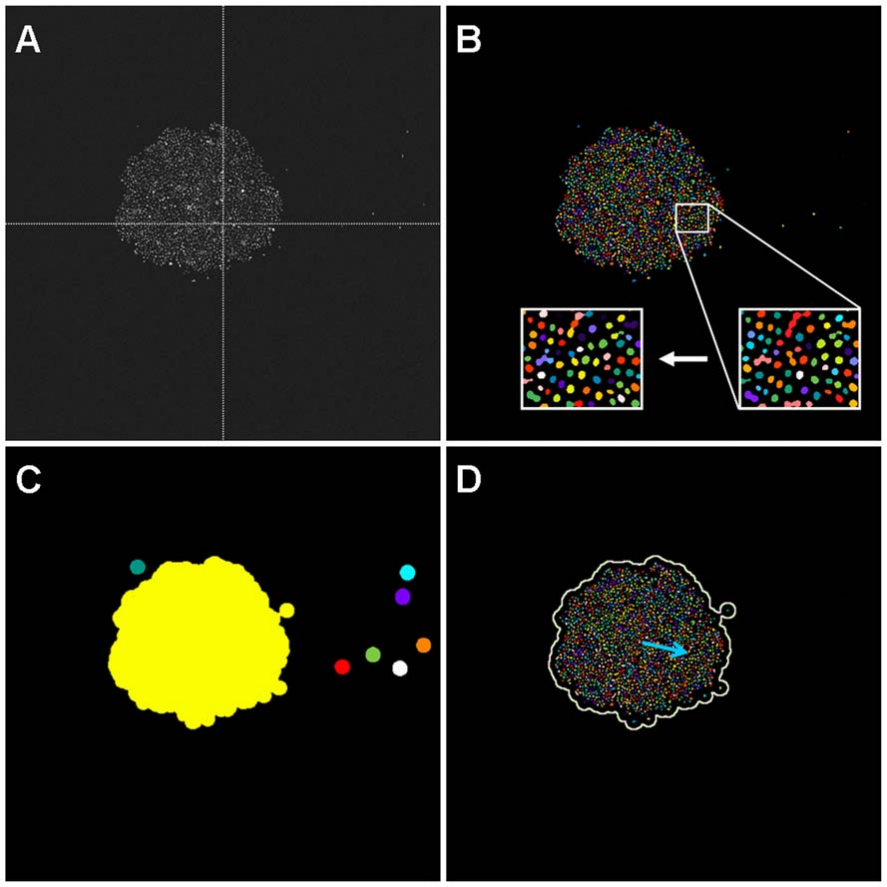
Image processing procedure to determine the cell count and dispersion values of a well. (A) Colony nuclei image of each well was obtained by stitching four adjacent, non-overlapping fields together. The example here shows a primary cell colony surrounded by several cell outliers. (B) Nuclei segmentation, which consists of a wavelet transform and watershed algorithm steps, was applied to identify all nuclei in the well. (C) The nuclei segmentation mask was then dilated to generate merging region areas where distinct cell clusters could be isolated. In general, the largest region (yellow), representing the cell colony of interest, and other smaller regions (other colors), representing outlier cell clusters, were identified. (D) Nuclei within the colony of interest were kept for measurement. Cell count was determined by the total nuclei count within the colony. Cell dispersion was determined by applying the spreading coefficient formula. The blue arrow represents a vector centered on the colony center with distance equal to the spreading coefficient.

The analysis routine developed for this screening assay is illustrated in Figure 2. For each well image, total cell numbers in the colony and a spreading coefficient value were measured through the image segmentation routine. The spreading coefficient is defined as the standard deviation of the cell positions in the colony relative to the center of the colony. By combining time-course images of T1 and T2, we obtained the derived measurements Cell Count Ratio (CCR) and Cell Dispersion Ratio (CDR), which correspond to the cell growth status and the cell migration/scattering status of each cell colony, respectively. The results generated from these two ratio parameters were used to determine the EMT inhibitory properties of the test compounds. We also analyzed the uniformity and robustness of the assay (positive and negative control well CDR uniformity plots shown in Figure S1). We validated that the CDR signal was robust in the screening assay, where intra-plate Z-factor was consistently above 0.5 between positive and negative control signals.

### Identification of potential EMT inhibitors in targeted compounds screen

**Figure 3:**
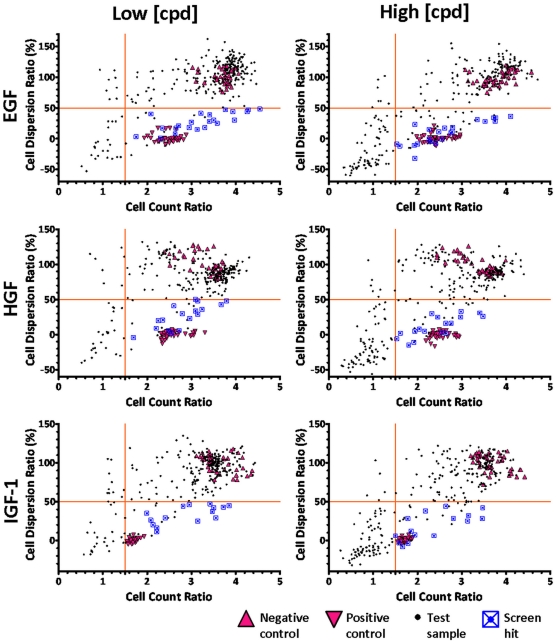
Cell dispersion ratio (CDR) vs. cell count ratio (CCR) plots. The graphs illustrate the behavior of NBT-II cells treated with different test compounds and growth factors in the spot migration assay. CDR threshold was set at 50% CDR between positive control CDR and negative control CDR. CCR threshold was set at 1.5 growth rate. We assessed compounds that inhibit cell dispersion (i.e. less than CDR threshold) and do not severely inhibit cell growth (i.e. more than CCR threshold). To further refine our hits, the test compounds were run at a low and high concentration (1.67 and 6.67 µM, respectively). Hit compounds (crossed squares) were classified as test compounds that satisfy the CDR and CCR threshold criteria at both concentrations.

We tested a collection of 267 targeted inhibitor compounds to determine if any of them could inhibit EGF-, HGF- or IGF-1-induced EMT in this screening assay (Figure 3). The complete data for the EMT targeted inhibitors screen is listed in Table S1. For hit finding, we were interested in identifying compounds that are not growth inhibitory (CCR ≥ 1.5) and could inhibit cell dispersion (CDR% ≤ 50%) with a wide concentration range more than 0.5 log differences. We therefore tested compounds at two concentrations (6.67 µM and 1.67 µM) in the EMT screen. Based on the screening data generated and the selection criteria that were set, we shortlisted 25 compounds that may potentially inhibit EGF-, HGF- or IGF-1-induced EMT.

**Figure 4:**
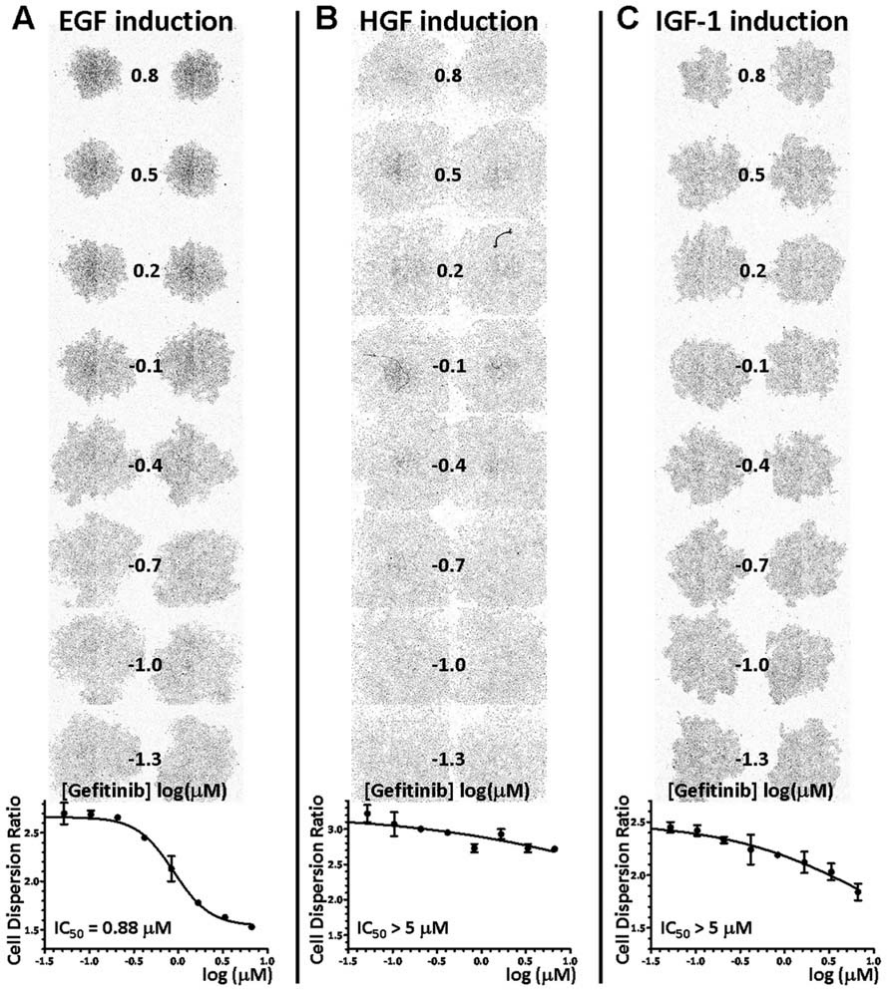
EMT inhibitory property of an EGFR inhibitor, Gefitinib. T2 plate image and CDR dose response profile of Gefitinib, against EGF- (A) HGF- (B) and IGF-1- (C) induced EMT.

**Figure 5:**
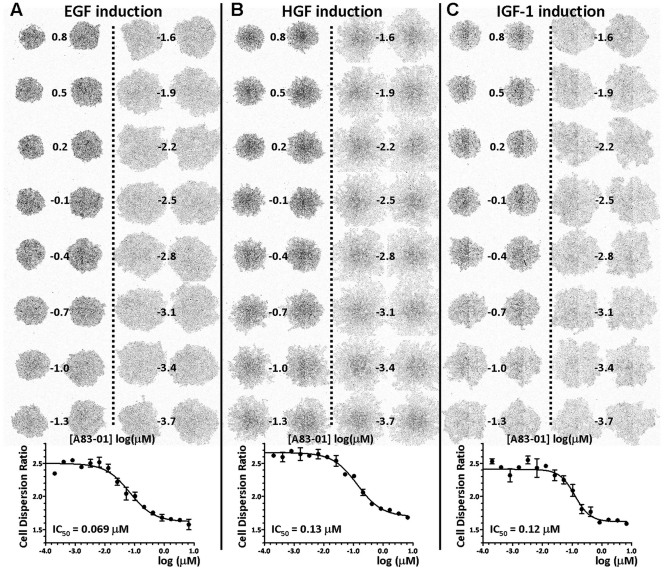
EMT inhibitory property of an ALK5 inhibitor, A83-01. T2 plate image and CDR dose response profile of A83-01, against EGF- (A) HGF- (B) and IGF-1- (C) induced EMT.

To determine the EMT inhibition potency of the 25 shortlisted compounds, we retested the compounds at diluting concentrations starting from 6.67 µM, using the same EMT spot migration assay for EGF, HGF or IGF-1 signaling. CDR dose response plots were generated for each compound/growth factor combination and the CDR IC_50_ values corresponding to the EMT inhibition potency were determined. We thus revealed compounds that were very effective against EMT by specific growth factors only (Figure 4). We also discovered compounds that were potent against EMT with all three growth factors (Figure 5). A summary of the CDR IC_50_ values for the 25 compounds is listed in Table 1. In general, we were able to group these compounds by their primary signaling molecules that they were designed to target. This grouping strategy helped us to validate this assay, where a c-Met inhibitor (e.g. PF-04217903) specifically inhibited HGF-induced EMT, while an EGFR inhibitor (e.g. Gefitinib) only inhibited EGF-induced EMT. We also identified four groups of compounds targeting ALK5, MEK, PI3K and SRC that were inhibitory to several EMT-inducing growth factors.

### Secondary assays to validate EMT inhibitors

We selected one compound from each target group and validated their EMT inhibitory response via time lapse video. We confirmed from the videos that an EGFR, c-Met and IGF-1R inhibitor could specifically inhibit EGF-, HGF- or IGF-1-induced EMT, respectively, as expected (Video S1, S2, S3). We also showed that ALK5, MEK, PI3K and SRC targeting compounds were indeed inhibiting migration induced by all three growth factors (Video S4, S5, S6, S7); this is interesting as these compounds are not the immediate and direct antagonists of the growth factors linked to EMT signaling.

**Table 1:** Summary of EMT inhibitor CDR IC_50_ values against EGF-, HGF- and IGF-1-induced EMT.

		Cell Dispersion IC_50_ (nM)			
Name	Target	EGF	HGF	IGF-1	Development stage^a^
JNJ-38877605	c-MET	>5000	94	>5000	Phase 1
PF-04217903	c-MET	>5000	55	>5000	Phase 1
AG1478	EGFR	330	>5000	>5000	Research
Erlotinib	EGFR	950	>5000	>5000	FDA approved
Gefitinib	EGFR	880	>5000	>5000	FDA approved
Lapatinib	EGFR	620	>5000	>5000	FDA approved
PD153035	EGFR	550	>5000	>5000	Research
PD158780	EGFR	1200	>5000	>5000	Research
WHI-P154	EGFR	800	>5000	>5000	Research
BMS-536924	IGF-1R, IR	>5000	2300	170	Research
A83-01	ALK5	69	130	120	Research
D4476	ALK5	1100	1400	1900	Research
LY-364947	ALK5	140	180	240	Research
SB-431542	ALK5	1600	940	820	Research
SD-208	ALK5	85	110	150	Research
AZD6244	MEK1/2	840	790	880	Phase 1-2
CI-1040	MEK1/2	1000	820	1200	Phase 2
PD0325901	MEK1/2	31	20	8.9	Phase 1-2
GDC-0941	PI3K	740	380	500	Phase 1
PI-103	PI3K	680	380	400	Research
PIK-90	PI3K	950	400	620	Research
ZSTK474	PI3K	850	410	660	Phase 1
API-2	PKB, AKT	1300	>5000	>5000	Phase 1
AZD0530	SRC, ABL	560	510	240	Phase 1-2
PP1	SRC	2300	2000	1200	Research

a Clinical trials information: ClinicalTrials.gov

We further investigated whether ALK5, MEK, PI3K and SRC targeting compounds could modulate the expression of EMT markers, such as E-cadherin and matrix metalloproteinase-13 (MMP-13) under EMT-activated conditions (Figure S2). With the exception of PI3K inhibitor GDC-0941, the compounds in general abrogated MMP-13 expression in growth factor-treated samples. PI3K inhibition had been previously shown to augment MMP-13 expression [23]. We also showed that PD0325901 and AZD0530 augmented E-cadherin expression in all three growth factor-treated conditions, while A83-01 and GDC-0941 restored the E-cadherin protein levels. These results suggest that selective inhibition of ALK5, MEK and SRC could potentially block EMT by restoring E-cadherin-mediated cell adhesion and reducing the invasion promoting MMP-13 and motility. These findings are consistent with previous reports showing that ALK5, MEK and SRC play a role in cell motility and tumor progression, while PI3K predominately regulates cell proliferation [24]-[26].

## Discussion

We have developed an EMT inhibition screening assay adapted for high-throughput/high-content screening of small molecule compounds. We have programmed a robotic liquid handler to deposit consistent, reproducible cell colonies as confined spots onto multi-well plates (Figure 1B). To the best of our knowledge, this method of confining cells to generate cell colonies within a few hours has not been previously attempted (the common alternative method is to allow sparsely attached cells to grow and form colonies, and this typically takes more than 3 days).

For image analysis, we use the wavelet transform and watershed segmentation methods [27]-[28] because the resultant nuclei segmentation is fast and accurate, suitable for high-content screening (Figure 2). We design the image acquisition and analysis method to encompass all cells in the well (Figure 1), rather than using representative snapshots of the well. This eliminates the issue of sampling bias, a common problem for high-content image analysis especially when cell distribution is not uniform. As this method accounts for all cells in the well, and the cell population per well is large (typically more than 1,000 cells), we expect that the analysis describing cell dispersion is reliable. In addition, because the entire cell population is analyzed, we are able to employ ratiometric analysis (i.e. comparing T2 and T1 images) to describe the growth of the cell colony (Cell Count Ratio) as well as to derive the colony dispersion over time (Cell Dispersion Ratio). We show that CDR values for the plate controls, which determine the upper and lower CDR boundaries, are consistent and robust (Figure S1), and this increases the confidence of the assay in hit determination.

The EMT spot migration assay has key advantages over traditionally described cell migration quantification methods, such as the Boyden chamber or the *in vitro* scratch techniques [29]-[30]. In general, these techniques are prone to sampling bias because, for practicality reasons, only representative microscope views and not the entire well image are chosen for analysis. Another key strength of the spot migration assay is that we are able to quantitate the cell proliferation and cell dispersion of each well simultaneously (Figure 2). This is important because we can then use this to select compounds that are predominately anti-migratory or EMT inhibitory against those that are generally toxic to cells (Figure 3), as cell toxicity will inherently hinder cell motility. Previous attempts to screen for cell motility modulating agents are mainly based on the *in vitro* scratch assay [30], which involves creating a scratch in a confluent cell monolayer and measuring the speed at which the cell layer grows or migrates to close this “wound”. Although the method has also been adapted for high-throughput screening by using robotic-driven pins to generate scratches on multi-well plates [31]-[32], it has failed to quantitatively differentiate whether the impairment of wound closure by the test agent is due to the inhibition of cell motility or the inhibition of cell growth pressure at the scratch front.

Our original motivation for designing this assay is to question whether targeted compounds previously selected and optimized to kill oncogene-addicted cells, can also be used to effectively inhibit EMT signaling. We have thus designed and validated a novel screening assay that can address the relative propensity and potency for small molecule compounds to block growth factor-induced EMT signaling. Through the primary screen and subsequent secondary assays, we have discovered that the druggable targets ALK5, MEK, SRC and to some extent PI3K may play a more significant role in EMT modulation and cancer progression, as their associated targeted compounds are inhibitory to several EMT-inducing growth factors (Table 1). The targeted compound library we assembled represents only a small subset of the targeted compounds that have been developed by universities and the pharmaceutical industry, and does not encompass all the druggable targets identified to date. Therefore, further extension of this EMT spot migration assay to include other diverse targeted compound libraries, such as the one assembled by Bamborough et al. [33], may allow us to discover other potent EMT inhibitors and EMT modulating targets.

However, the major limitation of this screening assay is that the reporter cell line NBT-II is not responsive to all exogenous growth factors that are known to induce EMT. For example, VEGF-A and VEGF-B have been reported to induce EMT in pancreatic and bladder cancer cells [34]-[35] but they do not induce a significant cell dispersion response in NBT-II (data not shown). In addition, although NBT-II serves as an efficient model to quantify the EMT inhibitory potential of compounds, the heterogeneous nature of carcinoma itself implies that not all the compounds that we have shortlisted here may inhibit EMT effectively as single agents in other cancer cell lines, where multiple EMT pathways may be switched on at the same time [13]. Thus, the selected compounds are currently being evaluated for their ability to revert the mesenchymal-like phenotype of carcinoma cell panels *in vitro* and in tumor xenograft models in immuno-compromised mice. We are also currently exploring the synergism effects between compound combinations [36] in inhibiting the EMT phenotype (example in Table S2). The objectives are to design new therapeutic modalities based on the EMT concept to interfere with tumor progression and to suppress resistance to chemotherapeutic agents [18], [37].

## Supporting Information

Figure S1 **Spot migration assay robustness.** Cell Dispersion Ratio of positive controls [6.67 µM AG1478 (A) 6.67 µM JNJ-38877605 (B) and 6.67 µM BMS-536924 (C)] and negative controls [0.67% DMSO] (A-C) for 3 experiment sets of triplicate plates (i.e. 8 data points per control condition per plate) is shown here for the spot migration assay against EGF (A) HGF (B) and IGF-1 (C) induction, respectively.
(PDF)

Figure S2 **Modulation of EMT markers, E-cadherin and MMP-13, by EMT inhibitors under EGF- (A) HGF- (B) and IGF-1- (C) induced EMT conditions.** E-cadherin expression levels decreased with growth factor addition compared with DMSO control, indicating cells had undergone EMT. However, the E-cadherin level was restored or augmented with increasing compound concentrations, due to EMT inhibition effected by these compounds. We observed two bands for E-cadherin, a 120 kDa band corresponding to the molecular weight of E-cadherin and a second band at 90 kDa that corresponded to a degradation form. Conversely, addition of HGF and IGF-1 increased MMP-13 expression levels compared with DMSO control. In general, this increase in MMP-13 expression could be abrogated with the addition of the EMT inhibitors. Positive control for each panel: 2 µM AG1478 (A) JNJ-38877605 (B) and BMS-536924 (C). α-tubulin was used here as a loading control.
(PDF)

Table S1 EMT spot migration data for 267 compounds tested at 1.67 and 6.67 µM concentrations, under EGF-, HGF- or IGF-1-induced EMT conditions. Data where CCR≤1.5 are highlighted magenta, indicating compound condition was growth inhibitory. Data where CDR%≤50% are highlighted green, indicating compound condition was dispersion inhibitory.
(PDF)

Table S2 EMT inhibition combination index (CI) values of ALK5 inhibitor A83-01 and c-Met inhibitor JNJ-38877605 combination against HGF-induced EMT. Cell dispersion ratio dose response profiles of A83-01 and JNJ-38877605 at fixed combinations ratios of 1:4, 1:2, 1:1 and 3:1 were generated using the spot migration assay. To determine if the EMT inhibitory effects obtained with different compound combinations were synergistic, we calculated the inhibition effect CI values according to the Chou-Talalay method using CalcuSyn software (Biosoft) (where CI>1.1, antagonism; CI = 0.9–1.1, additive effect; CI = 0.2–0.9, synergism; and CI<0.2 strong synergism). The results indicated that the combination treatment acted synergistically against HGF-induced EMT.
(PDF)

Video S1 Time lapse recording highlighting the EMT inhibitory response of the EGFR inhibitor, Gefitinib, under various EMT-inducing conditions. EGF only (A) HGF only (B) IGF-1 only (C) Gefitinib+EGF (D) Gefitinib+HGF (E) and Gefitinib+IGF-1 (F).
(AVI)

Video S2 Time lapse recording highlighting the EMT inhibitory response of the c-MET inhibitor, PF-04217903, under various EMT-inducing conditions. EGF only (A) HGF only (B) IGF-1 only (C) PF-04217903+EGF (D) PF-04217903+HGF (E) and PF-04217903+IGF-1 (F).
(AVI)

Video S3 Time lapse recording highlighting the EMT inhibitory response of the IGF-1R inhibitor, BMS-536924, under various EMT-inducing conditions. EGF only (A) HGF only (B) IGF-1 only (C) BMS-536924+EGF (D) BMS-536924+HGF (E) and BMS-536924+IGF-1 (F).
(AVI)

Video S4 Time lapse recording highlighting the EMT inhibitory response of the ALK5 inhibitor, A83-01, under various EMT-inducing conditions. EGF only (A) HGF only (B) IGF-1 only (C) A83-01+EGF (D) A83-01+HGF (E) and A83-01+IGF-1 (F).
(AVI)

Video S5 Time lapse recording highlighting the EMT inhibitory response of the MEK inhibitor, PD0325901, under various EMT-inducing conditions. EGF only (A) HGF only (B) IGF-1 only (C) PD0325901+EGF (D) PD0325901+HGF (E) and PD0325901+IGF-1 (F).
(AVI)

Video S6 Time lapse recording highlighting the EMT inhibitory response of the PI3K inhibitor, GDC-0941, under various EMT-inducing conditions. EGF only (A) HGF only (B) IGF-1 only (C) GDC-0941+EGF (D) GDC-0941+HGF (E) and GDC-0941+IGF-1 (F).
(AVI)

Video S7 Time lapse recording highlighting the EMT inhibitory response of the SRC inhibitor, AZD0530, under various EMT-inducing conditions. EGF only (A) HGF only (B) IGF-1 only (C) AZD0530+EGF (D) AZD0530+HGF (E) and AZD0530+IGF-1 (F).
(AVI)
