# Supplementary figures and images for: A Cell-Based Small Molecule Screening Method for Identifying Inhibitors of Epithelial-Mesenchymal Transition in Carcinoma
Source: PLoS One. 2012 Mar 14;7(3):e33183. doi: 10.1371/journal.pone.0033183 (PMC3303807; doi:10.1371/journal.pone.0033183)

Figure S1

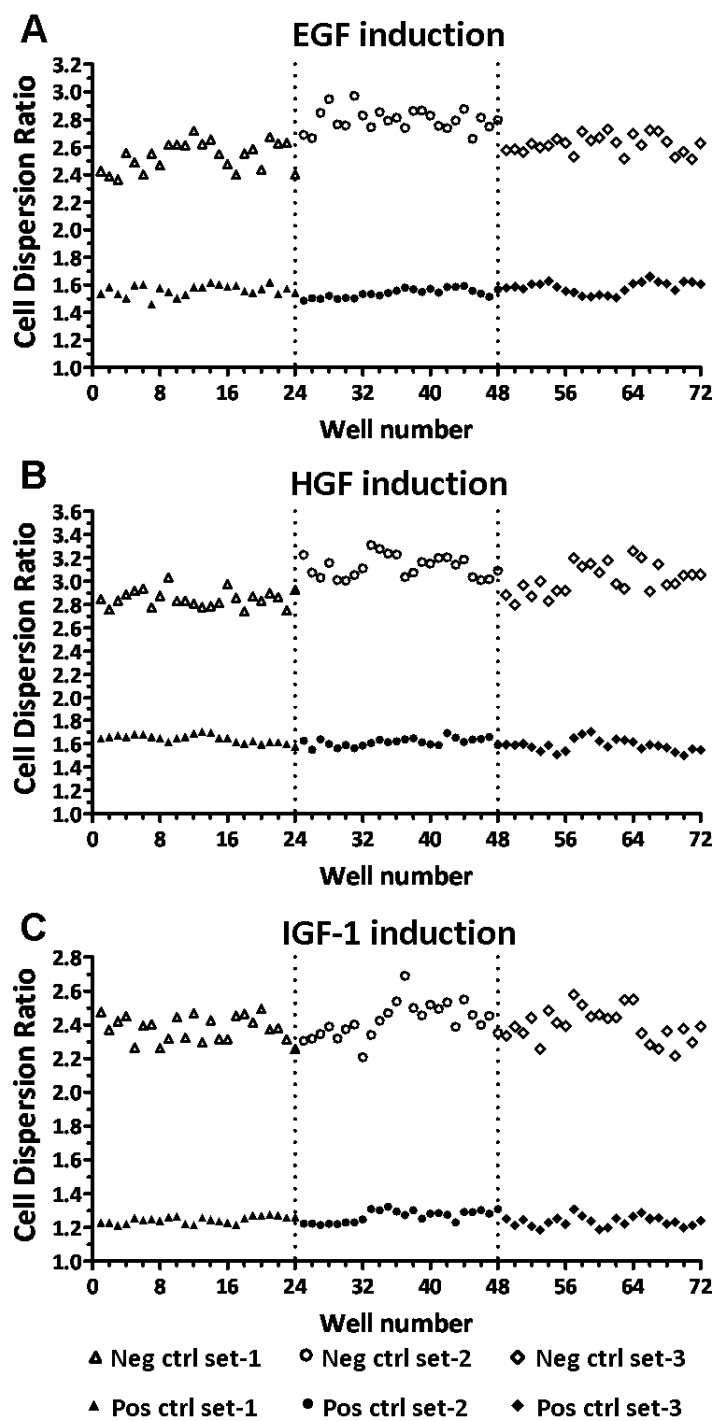

Supplement: Figure S1 — Spot migration assay robustness. Cell Dispersion Ratio of positive controls [6.67 µM AG1478 (A) 6.67 µM JNJ-38877605 (B) and 6.67 µM BMS-536924 (C)] and negative controls [0.67% DMSO] (A-C) for 3 experiment sets of triplicate plates (i.e. 8 data points per control condition per plate) is shown here for the spot migration assay against EGF (A) HGF (B) and IGF-1 (C) induction, respectively. (PDF) [file pone.0033183.s001.pdf]

Figure S2

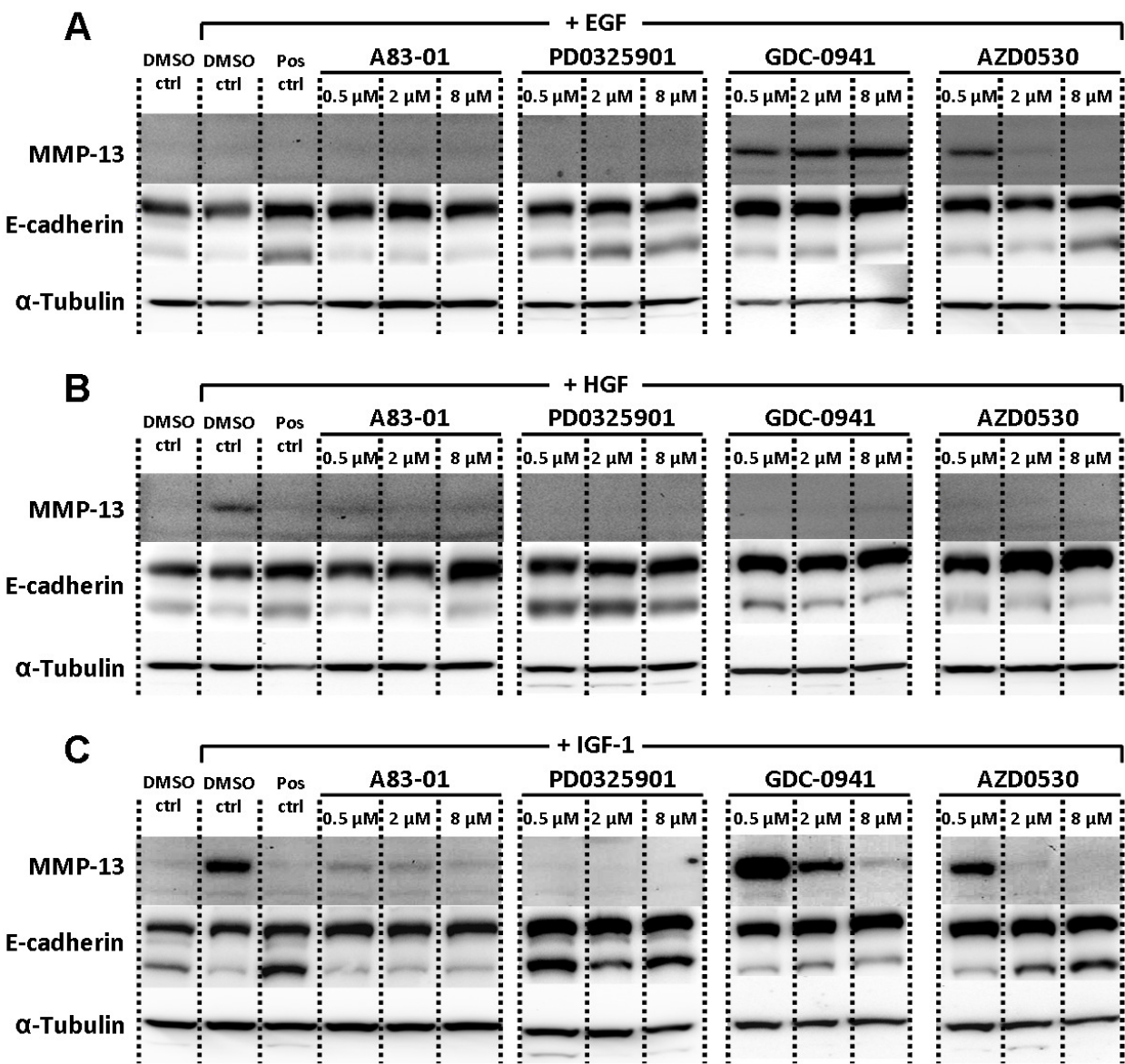

Supplement: Figure S2 — Modulation of EMT markers, E-cadherin and MMP-13, by EMT inhibitors under EGF- (A) HGF- (B) and IGF-1- (C) induced EMT conditions. E-cadherin expression levels decreased with growth factor addition compared with DMSO control, indicating cells had undergone EMT. However, the E-cadherin level was restored or augmented with increasing compound concentrations, due to EMT inhibition effected by these compounds. We observed two bands for E-cadherin, a 120 kDa band corresponding to the molecular weight of E-cadherin and a second band at 90 kDa that corresponded to a degradation form. Conversely, addition of HGF and IGF-1 increased MMP-13 expression levels compared with DMSO control. In general, this increase in MMP-13 expression could be abrogated with the addition of the EMT inhibitors. Positive control for each panel: 2 µM AG1478 (A) JNJ-38877605 (B) and BMS-536924 (C). α-tubulin was used here as a loading control. (PDF) [file pone.0033183.s002.pdf]
