# Supplementary material for: A Cell-Based Small Molecule Screening Method for Identifying Inhibitors of Epithelial-Mesenchymal Transition in Carcinoma
Source: PLoS One. 2012 Mar 14;7(3):e33183. doi: 10.1371/journal.pone.0033183 (PMC3303807; doi:10.1371/journal.pone.0033183)
Supplement: Table S1 — EMT spot migration data for 267 compounds tested at 1.67 and 6.67 µM concentrations, under EGF-, HGF- or IGF-1-induced EMT conditions. Data where CCR≤1.5 are highlighted magenta, indicating compound condition was growth inhibitory. Data where CDR%≤50% are highlighted green, indicating compound condition was dispersion inhibitory. (PDF) [file pone.0033183.s003.pdf]

Table S1

	EGF (20 ng/ml)				HGF (4 ng/ml)				IGF-1 (150 ng/ml)			
	Cpd (1.67 µM)		Cpd (6.67 µM)		Cpd (1.67 µM)		Cpd (6.67 µM)		Cpd (1.67 µM)		Cpd (6.67 µM)	
Compound Name	CCR	CDR%	CCR	CDR%	CCR	CDR%	CCR	CDR%	CCR	CDR%	CCR	CDR%
(-)-Terreic acid	4.2	113	3.1	73	3.7	78	2.8	73	3.5	97	1.9	68
(±)-Palmitoylcarnitine chloride	3.8	133	3.8	121	3.8	88	3.8	88	3.5	108	3.4	95
1,2,3,4,5,6-Hexabromocyclohexane	4.2	97	4.0	121	3.5	88	3.7	85	3.6	102	3.7	95
10-DEBC hydrochloride	4.1	96	1.0	-33	3.7	90	1.0	-10	3.3	102	0.8	-13
4-Chlorophenylguanidine hydrochloride	3.9	121	4.1	118	3.7	80	3.7	80	3.6	99	3.5	100
5-Azacytidine	2.9	94	2.0	45	3.4	87	2.2	25	3.2	59	2.4	51
8-Bromo-cAMP, sodium salt	3.9	123	4.2	114	3.8	87	3.9	96	3.6	109	3.5	92
A-3 hydrochloride	4.0	114	4.1	116	3.9	87	3.5	91	3.4	102	3.3	106
A83-01	4.0	30	3.8	34	3.1	29	3.0	25	3.5	37	3.1	32
ABT-702 dihydrochloride	4.0	112	3.6	71	4.1	87	4.0	55	3.5	92	3.4	63
ABT-737	3.9	105	4.1	96	3.4	109	3.7	112	3.3	101	3.5	107
ABT-869	3.7	78	2.0	46	3.1	108	1.7	48	4.1	95	1.7	29
ABT-888	3.6	82	3.3	82	2.4	115	2.3	109	3.9	99	3.7	102
AC 220	2.2	80	2.2	62	2.5	70	2.1	102	2.6	76	2.3	82
AG 13958	2.7	129	1.6	44	3.4	61	1.8	51	2.6	52	1.8	43
AG 1478 hydrochloride	2.7	21	2.5	11	2.8	67	2.8	65	3.0	72	3.0	68
AG 18	4.0	106	3.4	106	3.6	84	3.2	101	3.6	82	2.9	84
AG 213	4.0	131	4.0	100	3.9	89	4.1	101	3.6	88	3.4	84
AG 490	4.0	95	3.7	100	3.5	90	3.3	82	3.5	96	2.7	115
AG 494	4.2	106	3.3	110	3.8	94	3.0	95	3.6	93	2.1	109
AG 555	4.0	111	3.7	108	3.5	75	3.2	85	3.6	101	2.4	113
AG 556	3.9	126	3.7	107	3.5	86	3.1	80	3.3	118	2.8	91
AG 825	3.9	141	3.9	119	3.8	80	3.9	92	3.5	106	3.7	89
AG 99	3.9	144	4.1	137	3.9	95	4.1	105	3.6	102	3.7	96
Akt-I-1	3.8	90	3.8	102	3.4	92	3.3	96	3.3	103	3.3	86
Akt-I-1,2	3.4	88	2.9	44	3.0	102	2.5	107	4.0	102	3.4	69
AMG-47a	1.2	-33	1.0	-45	1.4	-30	1.1	-42	1.1	-7	1.0	-16
AMG-Tie2-1	4.2	97	1.8	-15	3.6	76	1.9	-9	3.5	94	1.8	11
Aminopurvalanol A	3.4	100	1.3	11	2.9	44	1.3	-20	2.6	37	1.1	-10
Anisomycin	0.9	-5	0.8	-23	0.9	4	0.8	-21	0.7	23	0.6	21
API-2	2.1	40	1.9	23	2.6	41	2.3	30	2.0	53	1.8	45
Apigenin	3.9	117	3.8	134	3.5	83	3.8	94	3.5	106	3.5	113
Arcyriaflavin A	4.1	118	3.6	127	3.6	81	3.7	87	3.5	101	3.5	94
AS-101	4.0	117	4.0	107	4.1	91	4.1	102	3.6	99	3.5	106
AS-252424	3.8	121	3.2	115	3.5	101	3.1	103	3.3	109	3.1	103
AT-7519	3.9	100	0.8	-48	2.7	107	0.8	-43	3.2	128	0.7	-15
AT-9283	1.1	-2	0.6	-47	1.1	2	0.5	-39	1.0	-2	0.7	-17
AV-412	0.8	-7	0.4	-59	0.7	-22	0.4	-47	1.1	61	0.5	-26
AV-951	3.0	99	1.1	61	2.0	126	1.1	43	3.6	116	1.2	38
Axitinib	1.1	96	1.1	96	0.7	115	0.9	106	1.2	104	1.2	86
AZ-960	1.2	-10	0.9	-40	1.1	3	1.0	-23	1.1	3	0.9	-14
AZD0530	3.1	15	1.9	-32	2.8	30	1.9	-11	2.2	11	1.7	-8
AZD2281	3.2	82	2.1	73	2.3	109	1.7	107	3.3	108	2.5	99
AZD6244	3.4	28	2.8	9	3.1	48	3.0	33	2.8	44	2.6	44
AZD7762	0.8	-13	0.7	-39	0.8	-20	0.7	-36	0.8	-15	0.6	-21
Bax channel blocker	2.9	109	0.4	-47	3.4	81	0.3	-45	2.8	78	0.3	-13
Bay 11-7085	2.9	78	1.1	-41	2.9	80	0.8	-25	1.9	55	1.0	-19
Bay 11-7821	3.0	78	1.1	-42	3.1	85	1.0	-39	1.7	53	1.1	-18
Belinostat (PXD101)	0.7	-27	0.7	-7	0.8	-13	0.6	-33	0.8	10	0.7	2
BEZ235	1.4	36	1.2	20	1.4	38	1.3	22	1.5	65	1.3	38
BI-2536 (R-)	0.9	-28	0.8	-37	0.8	-26	0.8	-11	0.9	-4	0.8	-9
BIBU 1361 dihydrochloride	3.3	35	0.6	-41	3.0	74	0.6	-31	3.0	74	0.9	-7
BIBX 1382 dihydrochloride	3.9	62	2.3	4	3.1	80	2.5	61	3.0	75	2.5	62
BI-D1870	2.4	73	1.1	-34	2.3	-1	1.0	-33	2.3	24	1.0	-12
Bisindoylmaleimide X HCl salt	2.9	128	0.5	-46	3.0	79	0.5	-37	3.2	78	0.6	-15
BMS-2	3.5	92	3.6	84	3.6	93	3.8	96	4.2	89	4.2	84
BMS-3	1.1	28	1.2	29	1.2	33	1.2	36	1.2	46	1.3	62
BMS-5	3.1	110	3.2	86	4.0	81	3.3	81	3.8	85	3.1	86

	EGF (20 ng/ml)				HGF (4 ng/ml)				IGF-1 (150 ng/ml)			
	Cpd (1.67 µM)		Cpd (6.67 µM)		Cpd (1.67 µM)		Cpd (6.67 µM)		Cpd (1.67 µM)		Cpd (6.67 µM)	
Compound Name	CCR	CDR%	CCR	CDR%	CCR	CDR%	CCR	CDR%	CCR	CDR%	CCR	CDR%
BMS-536924	3.6	82	3.0	42	3.6	89	2.6	24	2.1	26	1.9	7
BMS-599626	3.5	66	3.0	28	2.4	120	1.9	91	3.5	103	2.9	84
Bosutinib (SKI-606)	1.6	-27	0.8	-43	1.6	-21	0.9	-35	1.5	-3	1.0	-13
BX-795	1.1	78	1.0	34	0.8	108	1.0	37	1.1	105	1.1	103
BX-912	1.8	71	1.1	83	1.6	86	1.0	62	1.8	72	1.1	52
C-1	4.2	118	4.6	101	3.9	98	4.3	106	3.7	104	3.8	83
Camostat mesylate	3.8	96	3.6	90	2.6	124	2.3	99	3.9	112	4.1	103
Cantharidin	3.5	76	0.6	-47	3.6	70	0.7	-29	2.5	56	0.6	-19
CC-401	3.9	135	3.9	118	3.3	81	2.8	61	3.5	88	2.7	56
Cediranib (AZD2171)	2.6	81	0.8	-20	2.6	112	0.7	-27	3.9	87	0.8	9
Ceramide	4.0	120	3.6	131	3.8	93	3.7	93	3.5	111	3.3	98
Chelerythrine chloride	4.3	106	0.6	-49	3.9	92	0.3	-36	3.5	80	0.5	-32
CHIR-258	1.6	70	1.0	-12	1.7	49	0.9	-19	1.5	29	0.9	4
CI-1033	2.4	-12	0.9	-36	2.7	71	0.9	-7	2.8	56	0.9	17
CI-1040 (PD 184352)	3.5	30	2.3	-1	3.1	50	2.5	25	2.9	46	2.2	38
Clofarabine	1.7	60	1.3	14	1.6	52	1.3	46	1.4	56	1.0	35
CMPD-1	3.3	99	1.8	-1	3.3	74	2.0	4	3.0	87	1.4	2
CP-690550	4.0	109	3.7	109	3.5	81	3.5	78	3.5	108	3.7	106
CP-724714	2.6	89	2.4	62	2.6	98	2.6	105	3.4	83	3.2	75
CYC-116	0.9	112	1.1	31	1.1	4	1.0	-31	1.1	66	1.0	-4
Cyclopamine	4.0	126	3.9	129	4.0	88	3.7	89	3.5	102	3.4	115
Cyclosporin A	3.8	131	3.4	85	3.8	88	3.6	64	3.5	78	3.4	51
CYT11387	2.3	110	0.8	25	2.7	88	0.9	33	2.5	114	0.9	58
D4476	4.5	48	4.1	36	3.8	48	3.4	31	3.8	45	3.5	42
Dasatinib	1.3	-30	1.3	-41	1.3	-28	1.2	-33	1.1	-14	1.3	-16
Deforolimus	2.1	98	2.1	84	2.0	108	1.7	99	2.3	107	2.3	86
Demethylasterriquinone B1	3.7	144	1.3	-20	3.6	89	1.2	-15	2.9	110	1.2	-3
D-erythro-Sphingosine (synthetic)	3.9	128	3.8	125	3.5	78	3.6	80	3.6	92	3.7	87
Dibutyryl-cAMP, sodium salt	3.8	125	4.1	117	3.7	83	3.7	86	3.5	100	3.6	110
Dihydrosphingosine	3.9	142	4.0	118	3.7	87	3.8	91	3.5	98	3.5	98
DL-AP4	3.8	130	3.8	122	3.9	92	3.9	83	3.6	101	3.6	102
DMNB	4.1	125	4.0	112	3.5	87	3.8	91	3.6	108	3.6	95
DMPQ dihydrochloride	3.9	117	3.9	133	3.9	87	3.7	79	3.5	99	3.6	97
E7080	3.3	74	2.5	92	3.3	115	2.9	81	4.4	84	3.5	72
Enzastaurin	3.5	119	3.5	90	2.2	131	2.0	113	4.0	113	3.4	96
ER 27319 maleate	2.0	77	0.8	6	2.0	100	0.8	-17	1.8	91	0.7	-13
Erlotinib Hydrochloride	3.4	39	2.8	18	2.7	70	3.0	96	3.0	89	3.1	90
Fasudil hydrochloride	3.8	131	4.2	116	3.8	79	4.4	86	3.7	93	4.1	84
Flavopiridol	0.8	-15	0.8	-35	0.8	-19	0.8	6	0.7	-13	0.8	-11
FPA124	3.9	121	3.9	117	3.7	86	3.6	77	3.6	105	3.4	101
FTY720	0.6	-53	0.5	-56	0.6	-37	0.5	-39	0.6	10	0.5	-24
GDC-0941	3.0	18	1.9	-11	2.5	6	1.8	-15	2.2	16	1.6	-3
Gefitinib	3.3	18	2.6	-1	2.9	63	3.0	67	3.1	76	2.8	58
Genistein	3.9	137	3.1	129	3.6	87	3.4	84	3.3	105	2.9	100
GF 109203X	3.8	120	1.9	9	3.1	79	2.0	45	3.1	82	1.7	27
GSK1904529A	3.5	73	3.4	71	2.5	103	2.4	117	4.1	83	3.8	78
GSK690693	4.0	96	3.7	30	3.8	92	3.7	67	4.4	98	3.2	50
GTP 14564	4.3	106	4.2	119	3.7	89	3.7	89	3.7	98	3.6	82
GW 441756	3.9	143	3.1	127	3.6	83	3.1	54	3.2	86	3.1	49
GW 5074	4.1	128	4.5	106	3.6	100	4.1	91	3.7	102	4.0	93
GW 583340 dihydrochloride	4.2	53	3.3	30	3.0	88	2.8	76	3.1	78	2.5	80
H-7 dihydrochloride	4.2	137	4.4	111	4.2	91	4.3	97	3.6	98	3.9	111
H-9 dihydrochloride	4.1	130	4.2	130	3.7	91	3.8	90	3.6	111	3.9	103
HA 1100 hydrochloride	4.0	114	4.2	133	3.8	88	4.2	89	3.7	100	3.9	87
IC87114	3.8	111	3.9	94	3.6	84	3.6	88	3.4	107	3.1	116
Imatinib mesylate	3.8	107	3.5	100	2.4	121	2.5	128	3.0	124	2.7	118
IMD 0354	2.0	42	1.4	-41	2.2	84	1.2	-35	1.7	39	1.1	-17
INCA-6	3.9	121	3.4	80	3.7	80	3.0	85	3.4	96	2.4	79

	EGF (20 ng/ml)				HGF (4 ng/ml)				IGF-1 (150 ng/ml)			
	Cpd (1.67 µM)		Cpd (6.67 µM)		Cpd (1.67 µM)		Cpd (6.67 µM)		Cpd (1.67 µM)		Cpd (6.67 µM)	
Compound Name	CCR	CDR%	CCR	CDR%	CCR	CDR%	CCR	CDR%	CCR	CDR%	CCR	CDR%
Indirubin-3'-oxime	3.5	116	2.4	33	3.6	80	2.1	15	3.5	74	1.7	3
Indomethacin	3.8	121	4.1	129	3.7	81	3.7	89	3.6	108	3.4	102
Ionomycin free acid	4.1	113	3.6	102	3.9	94	3.2	117	3.3	92	2.8	89
JNJ28871063 HCl	3.1	79	3.2	44	2.3	118	2.3	106	3.4	107	3.2	86
JNJ38877605	3.8	107	3.5	110	2.6	2	2.6	3	3.4	77	3.4	72
JNJ7706621	1.6	64	1.1	1	1.4	70	1.1	40	1.7	93	1.0	19
K252a	0.8	-33	0.3	-47	0.8	-33	0.3	-44	0.7	-14	0.3	-23
Kenpaullone	4.2	132	3.0	15	3.8	73	2.8	24	3.7	67	2.8	21
Ki 20227	3.9	119	2.2	105	3.3	89	2.6	87	3.3	108	2.7	88
Ki 8751	1.8	100	0.9	21	1.5	111	0.9	70	1.7	97	0.9	27
KN-62	4.1	110	4.3	124	4.1	92	3.8	126	3.5	90	3.5	107
KT 5720	4.1	121	4.3	123	4.0	93	3.9	102	3.6	96	3.6	118
KT 5823	4.0	130	3.9	125	3.8	80	3.8	82	3.6	104	3.6	111
KU 0063794	1.7	58	1.5	37	1.9	87	1.5	63	2.2	48	1.5	61
KU 55933	3.2	84	2.3	45	3.1	102	2.1	128	3.8	90	2.3	68
Lapatinib Ditosylate	3.9	44	3.4	31	2.7	71	2.7	79	3.0	86	2.4	75
Lavendustin A	4.2	109	4.2	118	4.0	97	3.9	112	3.6	89	3.5	100
LFM-A13	3.9	136	4.0	143	3.7	96	3.9	99	3.4	102	3.5	94
L-NMMA acetate	3.9	125	4.3	120	3.7	77	3.9	89	3.6	96	3.6	116
LY 294002 hydrochloride	3.5	126	2.8	91	3.4	76	2.7	56	3.2	92	2.8	68
LY 303511	3.6	125	3.1	93	3.6	89	3.2	69	3.5	96	3.1	77
LY 364947	4.3	44	3.7	34	3.2	36	2.8	16	3.5	42	2.8	28
Masatinib	3.8	114	2.8	107	3.4	88	2.2	109	3.1	107	2.4	102
Merck-5	2.1	109	1.2	61	1.9	64	1.0	15	2.1	77	1.1	38
Metformin (1,1-Dimethylbiguanide HCl)	3.7	89	3.7	85	2.5	121	2.3	116	4.0	107	4.0	105
Methyl 2,5-dihydroxycinnamate	3.6	133	2.9	87	3.1	74	2.7	70	2.7	68	2.4	62
MK-2206	3.8	73	2.7	35	3.4	109	2.8	53	3.8	104	2.7	44
ML 9 hydrochloride	4.0	117	3.6	92	3.6	87	3.9	86	3.5	100	3.2	92
Monensin	2.4	11	1.0	-39	2.9	21	1.1	-32	2.6	30	1.1	-8
Motesanib	3.9	115	4.1	117	3.1	88	2.6	117	3.1	113	3.0	115
MS-275	2.2	33	1.1	-27	2.1	101	1.2	-10	2.2	60	1.2	7
Nigericin	1.4	-30	0.5	-49	2.2	-1	0.6	-41	1.5	-3	0.6	-12
Nilotinib	3.8	112	2.8	64	2.4	115	2.8	105	2.8	135	2.6	94
NPC 15437 dihydrochloride	4.1	106	3.2	59	4.1	105	3.9	79	3.3	98	3.1	51
NSC 625987	4.3	116	3.8	138	4.0	98	3.9	91	3.6	97	3.6	94
NSC 693868	4.0	130	4.1	124	3.6	89	3.9	92	3.4	96	3.7	98
Olomoucine	4.2	133	4.2	127	3.7	90	3.8	92	3.6	95	3.7	117
Pazopanib HCl	3.4	80	1.1	0	2.6	125	1.0	26	3.0	99	1.1	31
PD 0325901	2.3	1	2.0	-9	2.2	20	2.0	8	2.0	35	1.8	28
PD 153035 hydrochloride	2.6	8	2.4	-8	2.8	64	2.6	38	3.0	75	2.8	52
PD 158780	3.2	41	2.3	15	2.9	76	2.7	64	3.1	78	2.5	59
PD 173955	2.0	-11	0.9	-40	2.3	10	0.9	-37	1.8	8	0.9	-13
PD 173955-Analog1	2.1	53	1.1	-35	2.8	92	1.3	-34	2.3	83	1.2	6
PD 198306	3.7	51	1.8	-15	2.9	51	1.9	9	2.9	51	1.6	25
PD 407824	1.5	-8	1.2	-27	1.5	-5	1.1	-24	1.4	4	1.0	-9
PD 98059	4.4	105	4.5	98	3.8	80	3.6	70	3.8	84	3.5	66
Perifosine	3.5	88	3.5	82	2.6	115	2.3	113	4.1	119	0.9	-9
PF-04217903	3.8	97	3.8	112	2.7	6	2.7	2	3.5	58	3.5	73
PF-2341066	3.3	85	1.1	37	2.9	9	1.1	-21	3.5	68	1.1	17
PF-431396	1.3	99	1.1	2	1.2	33	1.0	-6	1.2	49	0.9	1
PF-562271	2.0	83	1.3	18	2.1	64	1.1	16	2.2	68	1.0	13
PHA665752	3.7	104	1.0	-59	2.3	4	1.0	-56	3.3	65	0.7	-31
Phorbol 12-myristate 13-acetate	2.1	110	2.3	107	1.6	95	1.6	106	1.6	119	1.8	105
PI-103	1.8	3	1.6	-12	1.7	-4	1.5	-6	1.5	9	1.4	-2
Piceatannol	4.0	97	3.8	126	3.8	74	3.6	94	3.7	90	3.3	86
PIK-75	0.5	-45	0.5	-45	0.5	-40	0.5	-42	0.6	-18	0.5	-18
PIK-90	2.6	17	2.6	17	2.3	21	2.2	5	2.4	29	1.9	12
PIK-93	3.1	74	2.1	54	3.3	79	2.5	45	2.2	107	1.9	59

	EGF (20 ng/ml)				HGF (4 ng/ml)				IGF-1 (150 ng/ml)			
	Cpd (1.67 µM)		Cpd (6.67 µM)		Cpd (1.67 µM)		Cpd (6.67 µM)		Cpd (1.67 µM)		Cpd (6.67 µM)	
Compound Name	CCR	CDR%	CCR	CDR%	CCR	CDR%	CCR	CDR%	CCR	CDR%	CCR	CDR%
PLX4720	3.7	98	4.0	73	2.4	110	2.9	99	2.7	110	2.9	86
PP 1	3.8	47	2.8	11	3.1	34	1.9	5	3.1	25	1.8	-4
PP 2	3.5	98	2.8	20	3.4	55	2.3	1	3.4	47	2.4	6
PP 3	3.9	125	3.9	116	3.7	86	3.6	85	3.4	109	3.6	100
PQ401	3.8	107	2.8	74	3.8	77	3.3	75	3.6	96	2.9	105
Procainamide HCl	3.7	92	3.7	87	2.9	122	2.3	120	4.1	105	3.9	111
Procaine HCl	3.8	87	3.7	93	2.6	127	2.4	104	4.0	118	3.9	115
Prostaglandin E2	4.0	117	4.1	104	3.7	80	3.7	84	3.5	100	3.6	109
Purvalanol A	3.9	127	2.3	37	3.8	78	2.2	-2	3.4	79	1.9	0
Purvalanol B	4.1	139	3.9	138	3.9	88	3.8	85	3.5	97	3.7	108
R1487	2.9	107	3.1	105	3.7	56	3.6	50	3.8	72	3.0	61
R59-022	3.9	102	4.0	105	3.8	86	3.8	78	3.5	99	3.5	85
Rapamycin	2.7	96	2.8	98	2.4	52	2.5	63	2.0	74	2.0	85
Resveratrol	3.8	121	4.4	126	3.9	92	3.9	90	3.6	99	3.6	112
Retinoic acid	4.0	99	4.1	103	3.4	72	3.4	70	3.3	69	3.4	82
RG108	3.4	110	3.6	98	2.3	113	2.3	111	3.8	117	3.9	107
RHO-15	4.4	110	4.0	115	4.0	101	4.0	98	3.7	114	3.7	117
Ro 08-2750	3.6	127	1.6	36	3.2	88	1.6	49	2.8	95	1.1	22
Ro 106-9920	3.1	98	1.1	-43	2.6	94	0.9	-40	1.8	62	0.9	-20
Ro 31-8220 mesylate	1.1	-20	0.9	-43	1.0	0	0.9	-36	0.8	-8	0.8	-19
Rottlerin	3.4	162	1.5	-20	2.0	121	1.4	-5	2.7	107	1.3	-9
RWJ-67657	2.9	112	3.1	114	3.2	53	3.1	43	3.2	66	3.1	46
Ryuvidine	1.1	-31	0.8	-43	1.1	-19	0.7	-42	0.7	-18	0.7	-19
SB 1518	2.1	65	0.5	-52	1.9	94	0.5	-46	2.2	45	0.6	-23
SB 202190	3.2	132	2.8	147	2.4	117	2.1	109	2.7	129	2.6	111
SB 203580	3.3	135	2.8	155	2.7	107	2.1	118	3.0	115	2.6	115
SB 203580 hydrochloride	3.6	122	3.0	132	2.4	119	2.0	121	3.1	94	2.8	97
SB 216763	3.4	144	3.6	100	3.4	75	3.0	69	3.3	106	3.2	85
SB 239063	3.6	126	3.7	124	3.4	66	3.2	51	3.6	84	3.5	70
SB 242235	2.7	113	2.9	121	3.8	65	3.5	52	3.4	66	3.1	55
SB 415286	3.9	132	3.8	154	3.3	108	2.7	114	3.2	113	3.2	122
SB 431542	4.3	47	3.7	28	3.7	43	3.5	26	3.7	43	3.5	28
SB 590885	4.1	69	0.7	-45	4.0	94	0.6	-42	4.0	112	0.6	-15
SC-10	3.9	121	4.2	118	3.6	86	3.6	81	3.5	112	3.6	108
SC-9	4.0	123	4.6	111	3.8	81	3.8	84	3.5	100	3.5	106
SD-06	2.6	112	2.2	127	3.6	58	3.5	70	3.1	61	3.5	73
SD-169	4.0	115	3.9	115	3.5	89	3.1	88	3.3	107	3.4	97
SD-208	3.6	25	3.5	28	3.0	23	2.6	16	3.5	29	3.1	25
SKF 86002 dihydrochloride	3.6	124	3.8	134	3.7	74	3.4	69	3.6	98	3.7	91
SKI II	3.7	120	3.5	84	3.6	96	3.3	61	3.6	84	3.0	38
SL 327	3.9	103	3.7	38	3.5	61	3.2	45	3.4	62	3.2	50
SNS-032	1.0	-20	0.9	-37	1.0	-11	0.9	-30	0.9	4	0.9	24
SNS-314	1.1	64	1.1	16	1.0	92	1.1	54	1.1	61	1.1	41
Sodfrum 4-Phenylbutyrate	3.6	91	3.8	84	2.4	121	2.6	97	4.2	113	4.2	104
Sorafenib Tosylate	4.2	89	2.4	17	3.1	115	2.3	68	4.1	121	2.4	52
SP 600125	3.7	157	3.4	128	3.5	77	3.3	62	3.4	101	3.4	74
SR 3677	4.1	100	3.8	89	4.3	110	3.9	94	4.4	100	4.1	90
Staurosporine	1.8	108	1.0	51	1.4	124	1.0	78	1.1	86	0.7	35
STK368014 (Amicarbalide)	3.1	93	3.9	80	2.5	107	2.5	104	3.4	103	3.9	107
STO-609 acetate	3.8	111	4.3	126	3.7	82	3.8	86	3.6	103	3.4	120
SU 11274	3.3	93	1.3	37	3.5	60	1.4	-7	3.8	89	1.5	36
SU 16f	4.5	89	2.1	35	3.8	62	1.9	21	3.5	91	2.0	30
SU 4312	4.1	127	4.0	102	3.8	94	3.6	67	3.6	105	3.8	74
SU 5402	4.0	119	3.9	121	3.3	94	3.4	91	3.4	98	3.5	96
SU 5416	4.1	118	3.8	122	3.8	88	3.4	81	3.5	102	3.5	99
SU 6668	3.1	98	2.9	104	3.8	114	3.7	78	3.8	88	3.8	86
Suberoyl bis-hydroxamic acid	2.9	106	2.2	89	1.9	132	1.6	104	2.9	132	2.0	117
Sunitinib	3.5	99	0.9	-41	3.1	41	0.9	-38	2.5	29	1.1	-13

	EGF (20 ng/ml)				HGF (4 ng/ml)				IGF-1 (150 ng/ml)			
	Cpd (1.67 µM)		Cpd (6.67 µM)		Cpd (1.67 µM)		Cpd (6.67 µM)		Cpd (1.67 µM)		Cpd (6.67 µM)	
Compound Name	CCR	CDR%	CCR	CDR%	CCR	CDR%	CCR	CDR%	CCR	CDR%	CCR	CDR%
TAK-715	3.2	106	2.9	73	3.9	97	2.7	14	3.5	63	2.2	27
Tandutinib (MLN518)	4.0	110	3.7	96	3.5	95	3.3	118	3.2	113	2.9	95
TBB	3.8	114	4.2	113	3.7	82	3.6	91	3.4	99	3.4	109
TCS 359	4.4	123	3.3	72	3.9	82	3.4	84	3.9	98	2.6	83
Temsirolimus	2.0	62	1.9	51	1.8	97	1.7	91	2.2	100	2.1	70
TG100115	3.9	116	3.8	113	3.4	85	3.7	91	3.3	104	3.4	100
TG101209	1.3	28	0.6	-7	1.3	6	0.6	12	1.2	-1	0.6	-11
TG101209-Derivative 1	1.3	65	1.2	-9	1.2	39	1.0	-25	1.3	34	1.0	-7
TG101209-Derivative 2	1.2	28	0.9	-36	1.2	20	0.8	-13	1.2	11	0.9	-14
TG101348	1.8	34	0.7	-35	1.7	16	0.7	-9	1.6	8	0.7	-16
TGX221	3.6	106	2.9	102	3.3	80	2.7	106	2.7	115	2.5	122
TPCA-1	3.5	106	1.3	57	3.4	85	1.3	23	3.2	90	1.2	15
Tyrphostin B44, (-) enantiomer	3.9	131	3.7	102	3.8	98	3.2	102	3.3	114	2.7	106
Tyrphostin B44, (+) enantiomer	4.0	109	3.7	100	3.8	75	3.3	80	3.7	93	2.7	105
U0126	4.0	88	3.2	24	3.4	65	3.1	45	3.1	55	2.9	48
Valproic acid, Na salt	3.9	93	3.8	89	2.5	132	2.4	124	4.0	113	4.0	115
Vandetanib	2.9	122	2.9	11	3.0	65	2.8	44	3.0	65	2.6	32
Vargatef	3.1	38	0.5	-60	2.7	32	0.5	-53	2.1	6	0.5	-29
Vorinostat (SAHA)	1.1	18	0.7	-31	1.1	45	0.7	-6	1.1	50	0.7	-8
VX-680	1.3	111	1.3	102	1.0	73	1.2	64	1.2	75	1.2	34
VX-702	4.0	104	3.8	106	3.8	89	3.7	110	3.6	95	3.6	103
W-7 hydrochloride	4.0	116	3.8	108	3.8	91	3.1	112	3.5	100	3.2	92
WHI-P 154	3.0	27	2.3	-5	3.0	71	2.7	51	3.1	77	2.8	55
Wortmannin	3.5	94	3.4	103	3.5	93	3.0	91	2.9	93	2.5	84
XAV939	3.3	77	3.2	72	2.3	115	2.5	110	3.9	91	3.7	72
XL-147	3.6	94	3.4	72	2.5	122	2.6	118	4.1	97	3.8	85
Y-27632 dihydrochloride	4.3	95	4.0	104	4.2	104	4.2	115	3.6	84	3.6	92
YM 201636	3.8	97	2.5	20	3.3	76	2.3	3	3.0	93	1.4	-6
ZM 306416 hydrochloride	3.9	68	2.4	-1	2.9	65	2.5	47	3.1	73	2.7	52
ZM 323881 hydrochloride	3.3	118	4.0	117	3.9	94	3.9	88	3.5	101	3.6	102
ZM 336372	4.4	124	4.7	96	3.3	119	4.0	111	3.5	110	3.6	100
ZM 39923 hydrochloride	4.1	133	3.9	119	3.7	83	3.5	93	3.5	106	3.5	95
ZM 447439	1.3	83	1.2	11	1.2	38	0.9	-16	1.2	62	1.0	15
ZM 449829	4.1	116	4.3	118	3.7	83	3.8	90	3.5	107	3.5	106
ZSTK474	2.3	17	1.5	-9	2.2	9	1.6	2	2.1	20	1.5	6
