# Supplementary material for: A Cell-Based Small Molecule Screening Method for Identifying Inhibitors of Epithelial-Mesenchymal Transition in Carcinoma
Source: PLoS One. 2012 Mar 14;7(3):e33183. doi: 10.1371/journal.pone.0033183 (PMC3303807; doi:10.1371/journal.pone.0033183)
Supplement: Table S2 — EMT inhibition combination index (CI) values of ALK5 inhibitor A83-01 and c-Met inhibitor JNJ-38877605 combination against HGF-induced EMT. Cell dispersion ratio dose response profiles of A83-01 and JNJ-38877605 at fixed combinations ratios of 1:4, 1:2, 1:1 and 3:1 were generated using the spot migration assay. To determine if the EMT inhibitory effects obtained with different compound combinations were synergistic, we calculated the inhibition effect CI values according to the Chou-Talalay method using CalcuSyn software (Biosoft) (where CI>1.1, antagonism; CI = 0.9–1.1, additive effect; CI = 0.2–0.9, synergism; and CI<0.2 strong synergism). The results indicated that the combination treatment acted synergistically against HGF-induced EMT. (PDF) [file pone.0033183.s004.pdf]

Table S2

A83-01 : JNJ-38877605 combination ratio	50% CI	75% CI
1:4	0.63	0.41
1:2	0.67	0.34
1:1	0.47	0.23
3:1	0.33	0.13
